# Developments in FRET- and BRET-Based Biosensors

**DOI:** 10.3390/mi13101789

**Published:** 2022-10-20

**Authors:** Yuexin Wu, Tianyu Jiang

**Affiliations:** 1 Shenzhen Research Institute of Shandong University, Shenzhen 518000, China; 2 State Key Laboratory of Microbial Technology, Institute of Microbial Technology, Helmholtz International Lab for Anti-Infectives, Shandong University-Helmholtz Institute of Biotechnology, Shandong University, Qingdao 266237, China; 3 School of Life Sciences, Peking University, Beijing 100871, China

**Keywords:** fluorescence resonance energy transfer (FRET), bioluminescence resonance energy transfer (BRET), biosensors, imaging, immunosensors, nanosensors, whole-cell sensors

## Abstract

Resonance energy transfer technologies have achieved great success in the field of analysis. Particularly, fluorescence resonance energy transfer (FRET) and bioluminescence resonance energy transfer (BRET) provide strategies to design tools for sensing molecules and monitoring biological processes, which promote the development of biosensors. Here, we provide an overview of recent progress on FRET- and BRET-based biosensors and their roles in biomedicine, environmental applications, and synthetic biology. This review highlights FRET- and BRET-based biosensors and gives examples of their applications with their design strategies. The limitations of their applications and the future directions of their development are also discussed.

## 1. Introduction

**Figure 1:**
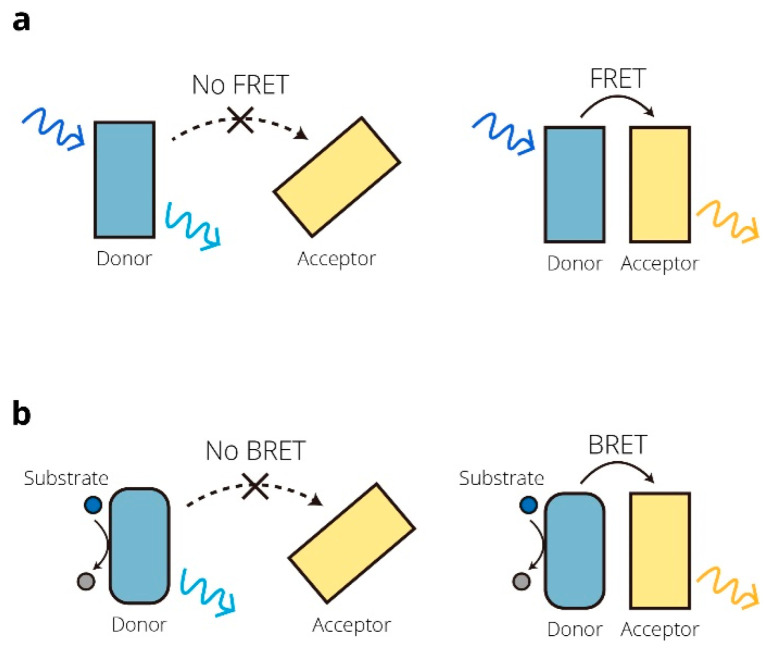
Principles of FRET and BRET. (**a**) Schematic representation of the FRET process, where the energy transfers from the donor to the acceptor in proximity. (**b**) Schematic representation of the BRET process. The donor luciferase oxidizes the substrate, then produces bioluminescence to excite the acceptor.

Since Theodor Förster raised an equation to quantify the excitation transfer efficiency from an energy donor to an acceptor in 1948 [1], technologies based on the resonance energy transfer (RET) mechanism have become a hit in diverse fields. RET is a nonradiative transfer of energy from a donor to an acceptor. When the donor is in close proximity to its acceptor, generally 10–100 Å apart [2], the energy from the donor can transfer to the acceptor. According to the energy donor, RET can be identified as fluorescence resonance energy transfer (FRET), bioluminescence resonance energy transfer (BRET), or chemiluminescence resonance energy transfer (CRET). FRET and BRET are widely used in research areas such as biology, medicine, and physiology. In FRET, the donor is excited by external light to emit fluorescence, and the energy is then transferred to the acceptor. In BRET, the donor is a luciferase that oxidizes the substrate luciferin to produce bioluminescence that excites the acceptor, causing light emission at a longer wavelength [3]. Principles of FRET and BRET are shown in Figure 1. RET-based biosensors based on distance and conformational changes are often developed for screening and imaging in various fields. The application of RET-based biosensors ranges from detecting various intracellular protein–protein interactions (PPIs) to reporting signal transduction pathways [4,5]. In addition, some RET-based biosensors are gradually integrated with optogenetics to contribute to the regulation of gene expression. As FRET and BRET technologies have continued to evolve, their applications in synthetic biology have been witnessed. In this review, an overview of the developments of FRET- and BRET-based biosensors, and their important roles in biomedical, environmental, and synthetic biology applications are summarized. We emphasize the design ideas and improvement approaches of newly developed biosensors. In addition, the limitations and the future directions of FRET- and BRET-based biosensors are also discussed.

### 1.1. FRET and FRET Systems

The Förster resonance energy transfer mechanism (also referred to as FRET) was proposed by Theodor Förster in 1948, who mentioned its possible use in biological studies [1]. Förster resonance energy transfer is a nonradiative (dipole–dipole) transfer of energy from a donor to an acceptor. Its efficiency is inversely proportional to the sixth power of the distance between the donor and the acceptor dipoles, while the relative orientation between the dipoles also affects FRET efficiency. When the donor is in close proximity to its acceptor, generally 10–100 Å apart [2], the energy from the donor can transfer to the acceptor. The transfer rate is not only linked to the distance of the donor and the acceptor but also depends on the overlap of the donor emission spectra and the acceptor absorption spectra, the donor’s quantum yield (QY), the absorption coefficient (ε_max_) of the acceptor, the relative orientation of the donor absorption, the acceptor transition moments, and the refractive index [6,7]. In the year 1967, Stryer and Haugland used naphthalene as the energy donor and a dansyl as the acceptor, and they were separated by oligomers of poly-L-proline (n = 1–12) by distances ranging from 12–46 Å. This structure formed a molecular ruler, suggesting that FRET could be used as a spectroscopic ruler in the 10–60 Å range in the study of biological macromolecules related to distance [8]. Such a study carried out by Stryer put the physics phenomenon FRET into biological use [9]. FRET has demonstrated its high temporal resolution and high sensitivity in its biological applications, where FRET was proved to be reliable in complex systems. However, it also has low resolution due to its own characteristics [10]. After the successful experiment with the molecular ruler, FRET grew in biological research. Fluorescent dyes and fluorescent proteins (FPs) are widely used in FRET systems. There are also studies with naturally occurring, intrinsically fluorescent biomolecules [11,12]. In addition to these, quantum dots (QDs), carbon nanotubes, graphene (oxide), carbon dots (CDs), metal complexes, metal nanoparticles (NPs), lanthanide-based up-conversion NPs (UCNPs), and fluorescent polymers have been included in studies on FRET [4,13,14]. The gene for the green fluorescent protein (GFP) derived from the jellyfish *Aequorea victoria* was first cloned [15] and expressed in other organisms [16,17,18]. This suggested that it is possible to use GFP mutants in FRET by genetically attaching donor and acceptor fluorophores to proteins [11,18]. Mutations and variants of GFP with different optical features, such as CFP, YFP, EBFP, EGFP, ECFP, EYFP, Citrine, Venus, DsRed, and mCherry, have been created and included in studies on FRET [18,19,20,21,22,23,24,25,26,27,28,29,30]. Early attempts to obtain FRET between GFPs all used BFPs as donors and phenolate anion GFPs, such as EGFP, as acceptors because these were the first available pairs with sufficiently distinct wavelengths. However, compared with CFPs, BFPs have disadvantages of poor extinction coefficients, quantum yields, and photostabilities. YFP is a classic acceptor with more spectrum overlaps and more distinct emissions when the donor is CFP [19]. To make the FRET pair CFP-YFP perform better, Nguyen and Daugherty developed an optimized CFP-YFP pair that displayed a 20-fold higher FRET signal ratio [31], which laid the foundation for commonly used FRET pairs. A number of mutations of GFPs have been created for screening the potential better FRET pairs [32]. In the past few years, QDs as well as other photosensitive nanomaterial bioconjugates play a role to reform the development of biosensors [33]. As new donors and acceptors have been discovered, different FRET systems have been developed.

### 1.2. BRET and BRET Systems

With the utilization of luciferase catalyzed bioluminescence, BRET overcomes the shortages of the fluorescence excitation of FRET, including light scattering and high background noise, and showed the possibility of measuring protein–protein interactions both in vivo and in vitro. The donor in the BRET system is luciferase, which oxidizes a substrate luciferin in the presence of O_2_ and sometimes a cofactor, such as ATP [11]. According to substrates, luciferases can be divided into different categories. Coelenterazine (CTZ or CLZ)-consuming luciferases, such as *Renilla* luciferase (RLuc) from *Renilla reniformis*, *Gaussia* luciferase (Gluc) from *Gaussia princeps*, *Oplophorus* luciferase (Oluc) from *Oplophorus gracilirostris,* and NanoLuc (Nluc) converted from a 19-kDa subunit of OLuc, frequently used CTZ or CTZ analog such as iphenylterazine (DTZ) and selenoterazine (STZ). NanoLuc is ATP-independent and bright, and has a small size of 19 kDa, making it one of the most widely used luciferases in recent studies. The commonly used substrate for NanoLuc is furimazine. There is evidence showing that furimazine has cytotoxicity at a high concentration [34]. This can be overcome by the development of furimazine derivatives [35]. The application of HaloTag in NanoBRET system makes it more convenient for acceptors with different spectral properties to be utilized in NanoBRET [36]. d-Luciferin-consuming luciferases contain the probably most widely researched and used luciferase for biology, firefly luciferase (FLuc or Flease in different studies) from *Photinus pyralis*. However, the reaction catalyzed by Fluc needs ATP and cofactor magnesium, which is different from CTZ-consuming luciferases. Along with its big size of 62 kDa and sensitivity to temperature and ionic strength, Fluc is not widely used in BRET like CTZ-consuming luciferases [37]. Bacterial luciferase from *Photorhabdus luminescens* has distinctive features. The autonomous bacterial bioluminescence system (Lux) can produce both luciferase and its long-chain aldehyde substrate [38] in a heterologous host. However, BRET pairs based on Lux system are rarely reported. Further progress should be made in BRET systems based on bacterial luciferase and other luciferases, such as *Vargula* luciferase (VLuc) from *Vargula* (formerly *Cypridina*) *hilgendorfi*.

In the year 1999, Xu et al. first used a BRET technique to study proteins encoded by circadian clock genes from cyanobacteria and demonstrated that the clock protein KaiB interacts with each other to form homodimers. RLuc was used as the donor in this study and the corresponding acceptor was EYFP, and the substrate was coelenterazine, respectively [39]. Later, to overcome the shortage of the traditional BRET (BRET1), new generations of BRET were developed including BRET2 [40,41], eBRET [42,43,44], BRET3 [41,45], BRET3.1 [46], BRET4 [47], BRET4.1 [46], BRET5 [46], BRET6 [46], BRET6.1 [46] BRET7 [47], BRET8 [47], and BRET9 [48], in which the donor, acceptor, and substrate in the bioluminescent reaction vary among systems to reach better effects (Table 1).

Some of the main pursuits are to prolong the detection timescale, improve sensitivity and stability, obtain a stronger near-infrared (NIR, wavelength range: 650–1700 nm) spectral signal for deep physiological tissue imaging to facilitate detection in biological samples [49], develop systems that are orthogonal to other systems, and diversify the colors of the signal, thus expanding the applications in broader application scenarios [50]. In addition to searching for suitable donors, acceptors, and substrates along the way, new methods for labeling the substance of interest efficiently with donors and acceptors are also hot topics in improving FRET and BRET.

## 2. FRET and BRET Strategies in Biosensors

It is of great significance in biosensors to understand the basic processes of life activities and to monitor these processes accurately. Biosensors can be used to measure products, metabolites, environmental factors, and metabolic fluxes to improve the precise understanding of related issues, and to monitor and control related processes. Recent years have witnessed myriad FRET- and BRET-based biosensors utilized in many areas, such as the diagnosis of diseases, in vivo imaging, and the detection of pollutants and pathogens. Biosensors can be classified according to the recognition element, such as antibodies, aptamers, enzymes, DNAs, MIPs, whole cells, etc. [51,52]. In addition, according to the analytes, biosensors can be classified into small molecules and ions, immune molecules, enzymes especially proteinases, nucleic acids, and tissue-based biosensors [53]. Ong et al. utilized BRET in high-throughput screening for the transient receptor potential vanilloid type 1 (TRPV1) ion channel [54]. Hu et al. used a single-color QD as the donor and two fluorescent dyes, Cy3 and Texas Red, as the acceptors in a FRET system for the detection of miRNA [55]. Circular templates specifically hybridized with target miRNAs were designed for the initiation of the hyperbranched rolling circle amplification (HRCA) reaction. The products hybridize with florescent reporter probes and capture probes then assembled on the surface of the QD, causing a different FRET signal that could suggest different miRNAs [55]. Yang et al. developed an N-CQDs/AuNCs nanohybrid-based FRET sensor for the detection of carbendazim, a broad-spectrum fungicide [56]. With the addition of carbendazim, the FRET interaction between carbendazim and AuNCs could recover the photoluminescence intensity of N-CQDs inhibited by AuNCs through FRET [56]. FRET and BRET have led to further advances in biosensor development, which is beneficial for providing diversified tools and platforms in biomedical, environmental, and synthetic biological studies.

### 2.1. Biosensors for Biomedical Research

#### 2.1.1. Biosensors for Bioassay and Diagnosis

FRET- and BRET-based biosensors are widely used for basic research on biological processes, such as signaling pathways, metabolism, and cell behavior, and to detect various substances of interest in organisms, both qualitatively and quantitatively. Biomarkers in the body, such as small molecules, nucleic acids, enzymes, proteins, antigens, hormones, metabolites, organelles, and cells, can be related to diseases and provide the possibility for precise and early diagnosis. Exosomes have been reported as valuable biomarkers associated with cancer-linked public health issues. For the quantitative detection of exosomes, Zhang et al. developed a self-standard ratiometric FRET nanoprobe, a Cy3-labeled CD63 aptamer (Cy3-CD63 aptamer)/Ti_3_C_2_ MXenes nanocomplex [57]. Without exosomes, the Cy3-CD63 aptamer could bind onto the Ti_3_C_2_ MXene nanosheets and FRET between the Cy3 and MXenes could cause the quench of the fluorescence signal from Cy3-CD63 aptamer. With added exosomes specifically combined with the aptamer and released from the surface of Ti_3_C_2_ MXenes, the fluorescence signal of Cy3 recovered. The hardly changed self-fluorescence signal of MXenes acted as a standard reference [57]. Krull et al. developed a FRET-based system for the sensitive screening of protein-based cancer biomarkers [58]. The aptamer-linked quantum dots (QDs-Apt) that could bind to the cancer biomarker protein epithelial cell adhesion molecule (EpCAM) was the donor and Cy3-labeled complementary DNA (cDNA) was the acceptor. With EpCAM competitive binding to QDs-Apt, the cDNA was displaced, resulting in the reduction of FRET [58].

In addition to biomarkers, other biological compounds, such as dopamine, have also attracted the attention of researchers. Liu et al. studied the secondary structure of a dopamine aptamer by isothermal titration calorimetry (ITC) and developed a biosensor for dopamine according to the resulting structure. A FAM fluorophore was labeled on the 5′-end and a dark quencher was labeled on the 3′-end at the edited aptamer DNA. With dopamine, the two ends come together, resulting in fluorescence quenching by FRET [59]. Singh et al. developed the first biosensor for the monitoring of isoleucine in living cells named the genetically encoded isoleucine indicator (GEII). To construct the nanosensor, they linked a periplasmic binding protein (LivJ) of *E. coli* with the FRET donor and acceptor, ECFP and Venus. In the presence of isoleucine, FRET between ECFP and Venus could be observed. The GEII shows potential for application in the metabolic engineering of high isoleucine yield bacteria [60]. Chen et al. screened an aptamer against polysialic acid (PSA), Apt3, and employed it in a sensitive FRET-based biosensor for PSA [61]. Calamera et al. reported a set of high-affinity FRET-based cGMP biosensors containing fluorophores with different optical properties. The biosensors were applied to detect cGMP produced through soluble guanylyl cyclase and guanylyl cyclase A in stellate ganglion neurons and guanylyl cyclase B in cardiomyocytes for intracellular signaling studies [62]. Glutathione (GSH) is related to redox and mediates a large variety of biological processes. Its abnormal levels are associated with human disease. Zhang et al. designed a multi-signal ICT-FRET probe Mito-CM-BP, which could detect GSH and its metabolite sulfur dioxide (SO_2_) simultaneously to visualize the metabolic processes of GSH to SO_2_ in living cells [63]. They developed a coumarin–cyanoacetic acid (CM) system to visualize GSH dynamics where CM acted as the donor of the FRET-I process and CM-GSH was the donor of the FRET-II process. The sensitive reaction site for SO2, benzopyrylium unit (BP), was the energy acceptor [63]. Crocker et al. developed AMPfret, a genetically encoded nanosensor for the cellular energy state where the donor and acceptor FPs were linked to N- or C-terminus of the AMP-activated protein kinase (AMPK) [64]. The binding of AMP or ADP to the γ subunit of AMPK could cause conformational change of the sensor, resulting in a FRET signal change. This FRET-based biosensor could detect changes in ATP/ADP and ATP/AMP ratios both in vitro and in cellulo [64].

**Figure 2:**
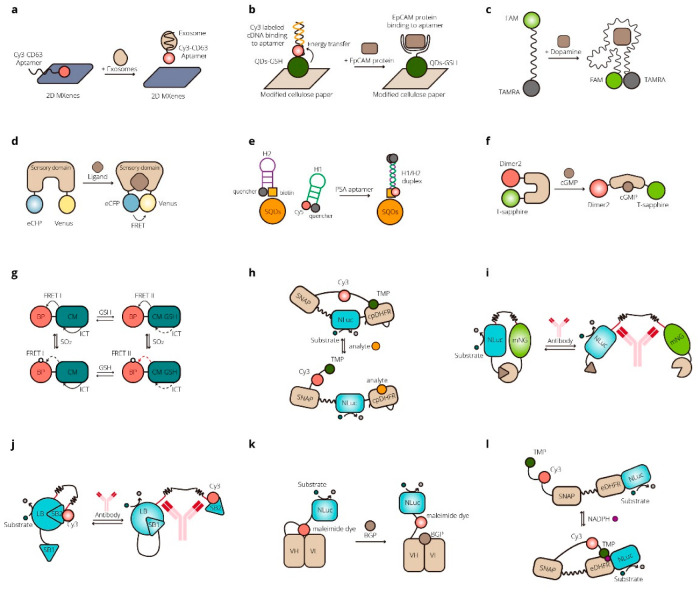
FRET- and BRET-based biosensors for biomedical applications. (**a**) A self-standard ratiometric, highly sensitive FRET platform for detecting exosomes [57]. (**b**) A FRET-based bioassay for the recognition of an epithelial cell adhesion molecule (EpCAM) [58]. (**c**) A folding-based FRET sensor for dopamine [59]. (**d**) A biosensor for the real-time optical tracking of isoleucine in living cells [60]. (**e**) A biosensor based on FRET and catalytic hairpin assembly for the detection of polysialic acid by use of a new DNA aptamer [61]. (**f**) A highly sensitive FRET biosensor for measurement of cGMP in cardiomyocytes and neurons [62]. (**g**) An ICT-FRET integration platform for the real-time monitoring of SO_2_ metabolism in cancer cells and tumor models [63]. (**h**) A BRET-based biosensor for point-of-care therapeutic drug monitoring [66]. (**i**) A BRET-based biosensor for detecting antibodies in blood plasma [67,68,69]. (**j**) A BRET-based biosensor for antibodies detection using intramolecular split luciferase complementation [70]. (**k**) A BRET-based immunosensor for antigens named BRET Q-Body [72]. (**l**) A BRET-based biosensor for NADPH [73].

The demand for portable, rapid, and sensitive detection at the point-of-care (POC) has grown for applications, such as early diagnosis of diseases and health monitoring of patients. RET-based biosensors, especially biosensors based on BRET where external light excitation is not necessary, have been put into POC applications. This progress has been well described by a comprehensive review [65]. One direction of POC applications is the quantitative detection of drug concentrations in the blood of patients. In 2014, a series of NanoLuc-based BRET-sensor proteins for the detection of small-molecule drugs (luciferase-based indicators of drugs, LUCIDs) were reported [66]. In designing the sensor, the anticancer agent methotrexate was chosen as the analyte. Bacterial dihydrofolate reductase (DHFR) was used as a receptor protein, and the DHFR inhibitor trimethoprim was the intramolecular ligand. The sensor was a fusion protein SNAP-Pro30-NanoLuc (NLuc)-cpDHFR linked to a synthetic molecule containing Cy3 and a DHFR inhibitor. With an analyte, the sensor could be shifted to an open conformation, reducing BRET efficiency. LUCIDs could monitor different drugs, such as the immunosuppressants tacrolimus and sirolimus, cyclosporin A, the antiepileptic topiramate, and cardiac glycoside digoxin [66]. RET-based biosensors have been applied for the detection of antibodies. In 2016, Arts et al. developed BRET-based single-protein sensors named LUMinescent AntiBody Sensors (LUMABS) consisting of a semiflexible linker between the donor NanoLuc and the acceptor green fluorescent protein mNeonGreen [67]. Helper domains keep the donor and acceptor close without the antibody of interest. When an antibody binds to epitope sequences flanking the linker, the interaction between the helper domains is disrupted, and the BRET efficiency decreases showing change in color from green-blue of the acceptor to blue of the donor. This provided the technology to easily measure picomolar antibody concentrations with a smartphone without the washing step. Not only can LUMABS recognize natural peptide epitopes, but they can also recognize nonpeptide epitopes. LUMABS sensors have been applied to the detection of antibodies against HIV1-p17, antibodies against hemagglutinin (HA), antibodies against dengue virus type I, dinitrophenol, creatinine, the Her2-receptor targeting trastuzumab, the anti-CD20 antibodies rituximab and obinutuzumab, and the EGFR-blocking cetuximab [67,68,69]. Based on LUMABS, Tenda et al. developed the first fully integrated microfluidic paper-based user-friendly analytical devices (μPADs) [70]. The BRET-based LUMABS was fixed into the device, which sensed the object of interest and produced color changes that could be captured by a digital camera. Researchers have provided opportunities for simultaneous detection of three different antibodies (anti-HIV1, anti-HA, and anti-DEN1) in whole blood in a highly user-friendly “just add the sample” manner [70]. By competitive intramolecular complementation of split NanoLuc, a new sensor format, NB-LUMABS, was reported in 2019 [71]. Two copies of a 1.3 kDa small BiT (SB) of NanoLuc were fused to either the N- or C-terminus of a single copy of an 18 kDa large BiT (LB) of NanoLuc to form a protein switch, while only SB on the N-terminus was conjugated to Cy3 for the emission of red light. Without antibody, the switch formed the conformation where the N-terminal SB binds to the LB and reconstitutes luciferase activity that allows for the BRET process to emit red light. This conformation can be disrupted by bivalent binding of an antibody resulting in nonfluorescently labeled SB combined with LB emitting blue light [71]. Takahashi et al. developed a BRET Q-body, in which luciferase NanoLuc is fused to a Q-body to construct a new immunosensor [72]. Quenchbodies (Q-bodies) are antibody fragments comprising an antibody fragment containing an antigen-binding site that is site-specifically labeled with a fluorescent dye. In this study, NLuc was fused to the N- or C-terminus of a single-chain antibody (scFv) fragment that specifically binds osteocalcin (bone Gla-protein, BGP) and then was labeled with ATTO520-C2-maleimide. In the presence of the antigen BGP-C7, the quenched fluorescent dye is released, and thus, NLuc oxidizes the luminescent substrate to provide energy transferring to the now available dye. A 12-fold higher response was acquired, implying that BRET Q-body is a useful biosensor in point-of-care tests [72]. Monitoring of biomarker metabolites is of great significance for the diagnosis, treatment, and management of numerous diseases. Yu et al. developed a biosensor that can measure NADPH by a digital camera in paper-based assays [73]. A fluorescently labeled ligand with NADPH-dependent affinity for the receptor is covalently tethered to the NADPH-dependent receptor protein through SNAP-tag. Without NADPH, the sensor is in an open state where the ligand could not bind to the receptor. The addition of NADPH could trigger the formation of the closed state where the binding of ligand and receptor brings the acceptor Cy3 close to NanoLuc, thereby increasing BRET. The NADPH concentration could be quantitatively calculated from the ratio of the emission intensities of NanoLuc and Cy3. This biosensor was applied for assay for phenylketonuria (PKU) with whole-blood samples [73]. Li et al. developed a paper-based BRET system for the analysis of tumor-associated circulating microRNAs (miRNAs) in clinical serum samples [74]. Some examples for FRET- and BRET-based biosensors for bioassay and diagnosis are shown in Table 2. And Figure 2.

#### 2.1.2. Biosensors for In Vivo Imaging

FRET- and BRET-based biosensors are widely used in in vitro and in vivo imaging and the analysis of biological compounds, making it possible to visualize various biological processes.

Yang et al. developed a BRET-based genetically encoded Ca^2+^ sensor that does not need external excitation, coordinating it with optogenetics techniques [75]. The troponin C domain (TnC) was inserted between C-terminal truncated Venus and NanoLuc luciferase. In the presence of Ca^2+^, the conformational change of the Ca^2+^-sensitive troponin sequence brought NanoLuc closer to Venus so that BRET could occur, resulting in a concomitant spectral shift. They put the sensor into quantifying and imaging Ca^2+^ fluxes elicited by brief pulses of light to cultured cells expressing melanopsin and to the neurons-expressing channel rhodopsin. The utilization of BRET sensors that do not need external excitation helps to eliminate undesirable consequences of fluorescence irradiation [75].

Adenosine 3′,5′-cyclic monophosphate (cAMP) is an important second messenger regulating plenty of intracellular functions. A classic example of studying the cAMP signaling pathway in cells is the sensor FlCRhR, which was the first FRET-based biosensor for cAMPs. It consists of cAMP-dependent catalytic subunits of protein kinase A I (PKA I), in which the catalytic (C) and regulatory (R) subunits are each labeled with fluorescent dye fluorescein or rhodamine, and FRET is shown in the holoenzyme complex R_2_C_2_. The C subunits could dissociate from the complex with cAMP bound to the R subunits, and the energy transfer is thus reduced [76]. However, chemically labeled proteins are unstable and hard to produce. They have to be microinjected into cells instead of expressed in cells, which could affect cAMP kinetics [77]. The traditional fluorescein- and rhodamine-labeled FlCRhR sensor was modified with BFP and GFP by Zaccolo et al. in 2000 [78], and then with CFP and YFP in 2002 [79]. These works make FlCRhR genetically encoded, and microinjection is not needed. The use of FPs allows for the elucidation of the biochemistry of cAMPs in vivo. FlCRhR was put into use for several different applications related to cAMPs, such as imaging cAMPs in neurons and neuronal networks [80] and the study of cAMP dynamics in oocytes [81,82]. Nagai et al. reported a cAMP-responsive tracer (ART) for visualizing the phosphorylation of proteins in living cells in 2000. It was the first FRET biosensor for protein kinases. Two GFP variants, RGFP and BGFP, were linked through the kinase-inducible domain (KID) of the transcription factor cAMP-responsive element binding protein (CREB). With PKA phosphorylation, the FRET among the flanking GFPs decreased [83]. For the detection of ATP, a BRET sensor was created by Min et al. named ARSeNL, ATP detection with a ratiometric mScarlet-NanoLuc sensor [84]. This biosensor employed a combination of NanoLuc and mScarlet as the donor and acceptor and showed a large dynamic range in detecting ATP. It was proposed that the development of ARSeNL could expand the toolbox of in vivo imaging of the metabolic status [84]. Shcherbakova et al. used a new red-shifted monomeric NIR fluorescent protein, miRFP720, to construct a FRET pair of miRFP670–miRFP720 for multiplexed imaging and light control of the Rho GTPase signaling pathway [85]. The development of miRFP670–miRFP720 pair enabled the further design of biosensors compatible with CFP-YFP imaging and blue-green optogenetic tools in use [85].

FRET- and BRET-based biosensors have been applied for in situ dynamic tumor microenvironment visualization. Zhao et al. reported on serial pH_t_ adjustable sensors (pTAS) for tumor pH monitoring [86]. The higher sensitivity and wider response region of these sensors were achieved by regulation of the component ratio of the second near-infrared (NIR-II) emission aza-BODIPY (NAB) donor and pH-sensitive rhodamine-based pre-acceptor (NRh). The sensor achieved dynamic visualization of in vivo tumor pH change processes through dual-channel ratiometric bioimaging within the NIR-II window [86].

The imaging of cells and tissues is a vital aspect of applications. Taylor et al. designed a BRET reporter based on NanoLuc and LSSmOrange in combination with FLuc and ZsGreen [87]. Two BRET processes with distinct characteristics allowed for highly sensitive visualization of different cell populations in vivo within the same imaging session. It also facilitated image signal overlay and the identification of areas of colocalization [87].

### 2.2. Biosensors for Environmental Applications

FRET- and BRET-based biosensors are used in the detection of toxic small molecules in food and the environment. Tang et al. designed a nanobody-mediated immunosensor based on FRET between different-sized QDs [88]. QDs of two sizes were covalently labeled with OTA and Nb, acting as the energy donor and acceptor. Both the free OTA and the donor could bind to the acceptor. When OTA concentration increased, the FRET efficiency decreased for less donor bonded to the acceptor. The sensor allowed rapid detection of OTA in agto-products in 5 min with a detection limit of 5 pg/mL [88]. Sabet et al. developed a FRET-based sensor for the detection of aflatoxin B1 (AFB1) [89]. The QDs conjugated with aptamer in the sensor were quenched via FRET without AFB1 due to the interaction of the aptamers with AuNPs. The aptamers were attracted to the added AFB1 from the AuNPs, and the fluorescence could recover. The developed biosensor was applied for the analysis of AFB1 in rice and peanut samples [89]. A series of biosensors based on FRET for the determination of organophosphate pesticides were designed by Wu et al. [90]. The fluorescence emission of carbon quantum dots (CQDs) could be quenched by AuNPs. Butyrylcholinesterase (BChE) could hydrolyze acetylthiocholine (ATC) to produce thiocholine, which could cause the aggregation of AuNPs and the corresponding recovery of FRET-quenched fluorescence emission. With the organophosphorus pesticides (OPs), the recovery of fluorescence in the sensor was reduced owing to the irreversibly inhibited catalytic activity of the BChE by the OPs. The biosensor was applied for OP detection in tap and river water samples [90].

Heavy metals are one of the environmental pollutants researchers aim to detect. For the detection of Hg^2+^, Li et al. developed a turn-on nanosensor based on FRET between long-strand aptamer-functionalized UCNPs and short-strand aptamer-functionalized gold nanoparticles (GNPs) [91]. The UCNPs were initially quenched due to the specific matching between the two aptamers. The stable binding interactions between Hg^2+^ and thymine could induce the long-stranded aptamers to fold back forming a hairpin structure. This caused GNPs to release from the UCNPs, and the fluorescent signal recovered. The sensor was applied to detect Hg^2+^ in tap water and milk samples [91]. Liu et al. employed gold nanorods (Au NRs) as the energy acceptor and CDs as the donor to construct a biosensor for Pb^2+^. The Au NRs were absorbed on the surface of the CDs, resulting in a quenched fluorescence signal of the CD–cysteamine–Au NR assembly. The Pb^2+^ ions bound completely with the cysteamine and disturbed the FRET process, and the fluorescence signal was restored. The sensor performed well in the detection of Pb^2+^ ions in samples of tap water and river water samples [92].

**Figure 3:**
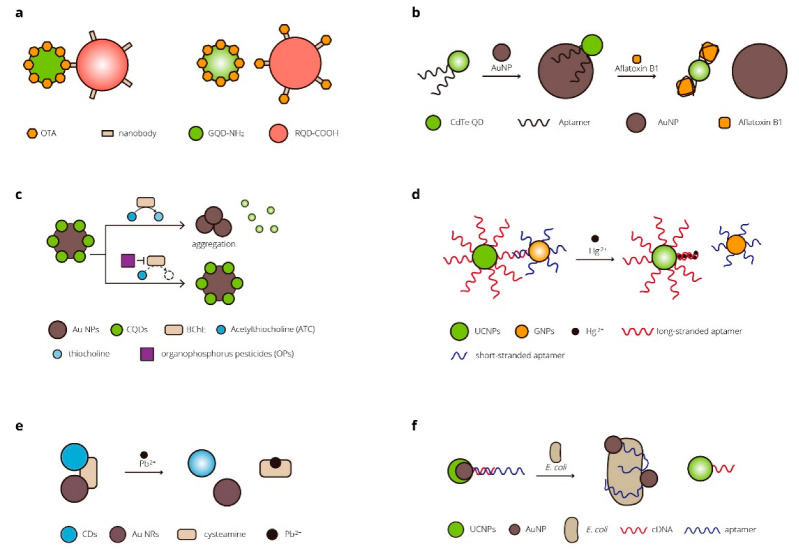
FRET- and BRET-based biosensors for environmental research. (**a**) A FRET-based immunosensor for the detection of ochratoxin A in agro-products [88]. (**b**) A FRET-based aptamer biosensor for the detection of aflatoxin B1 in peanut and rice [89]. (**c**) FRET-based sensors for organophosphate pesticide determination [90]. (**d**) A FRET-based biosensor for Hg^2+^ in food [91]. (**e**) A FRET-based biosensor for off-on detection of Pb^2+^ [92]. (**f**) A FRET-based aptasensor for rapid and ultra-sensitive bacteria detection [93].

In addition to pollutants, there is also concern about pathogenic microorganisms in the environment. Jin et al. developed a novel detection platform based on FRET for specific bacteria detection in the environment and food [93]. Upconversion nanoparticles (UCNPs) acted as the donor and were functionalized with the corresponding cDNA. The acceptor AuNPs that could cause fluorescence quenching were conjugated with aptamers. Without target bacteria, the aptamers bound to the cDNA, resulting in a quenching of the UCNPs. With the bacteria of interest, the aptamers preferentially bound to the bacteria rather than the cDNA to dissociate UCNPs–cDNA from the AuNP–aptamers, and the upconversion fluorescence would recover. The sensor was proved to efficiently detect *E. coli* in real food and water samples such as milk and tap/pond water within 20 min [93]. Table 3 and Figure 3 show some of the FRET- and BRET-based biosensors for environmental applications.

### 2.3. Biosensors for In Vivo Dynamic Analysis of Metabolic Flux

Metabolic flux is of great importance in metabolic engineering and facilitates the study of biosynthetic pathways. To precisely control the engineered system and obtain an improved metabolite flux, measuring the metabolites concentrations and flux rates and metabolic intermediates is a vital part of synthetic biology [95]. Many biosensors have been developed to address this problem, among which are RET-based biosensors (Table 4).

Engineering microbial strains to produce L-lysine draw scientists’ attention in industrial biotechnology. Thus, Ameen et al. developed a series of genetically encoded FRET-based nanosensors, namely FLIPK for the real-time monitoring of lysine at a cellular level [95]. The lysine binding periplasmic protein (LAO) from the *Salmonella enterica serovar typhimurium* LT2 strain was utilized as a part of a reporter, which was sandwiched between CFPs and YFPs. They tested the sensors for successfully monitoring the intracellular level of lysine both in bacterial and yeast cells and concluded that the sensors can be applied for the in vivo measurement of lysine levels in eukaryotes as well as prokaryotes. The sensors could be further used to measure real-time intracellular lysine levels in metabolically engineered microbial strains [95].

In order to analyze the metabolic flux of the (+)-catechin biosynthetic pathway, Kausar et al. developed a fluorescence indicator protein named FLIP-Cat, a FRET-based nanosensor for in vivo real-time monitoring of the metabolic flux of the (+)-catechin [97]. This genetically encoded nanosensor was composed of a (+)-catechin binding protein fraa-3 from *Fragaria ananassa* as a ligand-sensing domain, ECFP as the donor, and Venus as the acceptor. The donor ECFP was linked to the N-terminus of the fraa-3 protein, and the acceptor YFP was fused to the C-terminus of the fraa-3 protein. With the binding of the (+)-catechin, the fraa-3 protein underwent conformational changes for FRET to occur. The researchers then designed a (+)-catechin biosynthesis pathway and introduced it in *E. coli* along with the biosensor FLIP-Cat. With addition of different substrates, they measured the metabolic flux of the (+)-catechin in real-time and identified that dihydroflavonol reductase (DFR) was the main regulatory element. DRF can be utilized for controlling the (+)-catechin biosynthetic pathway, thus enhancing the production of catechin [97]. FRET- and BRET-based biosensors have good performance in monitoring metabolic processes according to the previous study. This could increase researchers’ understanding of specific processes and help researchers engineer metabolic processes, which is of great significance in synthetic biology research related to metabolic engineering.

## 3. Conclusions and Prospects

Biosensors based on FRET and BRET are widely utilized in environmental, medical, and biological research. FRET- and BRET-based biosensors have flourished most in biomedicine. Many biosensors have been applied for biomarker detection and point-of-care detection. They are also applied in the detection of pollutants and pathogenic microorganisms in environmental research. In addition, FRET- and BRET-based biosensors have also been applied to applications in synthetic biology, such as monitoring metabolic flux. The match between the viable range of resonance energy transfer and the appropriate length scale of biomacromolecules, as well as its explicit technology mechanism, and its greater accessibility in sample preparation and measurement conditions than some other non-optical techniques make FRET- and BRET-based biosensors more widely used [4]. Designing FRET- and BRET-based biosensors with superior performance for application remains challenging. FRET is criticized for its signal-to-noise ratio, inadequate fluorescence resolution, low stability of reagents, etc. BRET sensors do not need external light for excitation, thus overcoming problems such as the signal-to-noise ratio, autofluorescence, and photobleaching. However, the addition and delivery of the substrate and possible cytotoxicity are of concern. More efforts should be made to design BRET-based sensors for higher sensitivity and a high signal-to-noise ratio. Over past years, many researchers have devoted their efforts to finding more suitable donors, acceptors, and substrates that meet specific research needs, such as non-invasive deep tissue imaging and reversible real-time detection. However, the journey still has a long way to go. Multiplexed-RET system for the simultaneous detection and analysis of multiple analytes is another method of improvement. Additionally, efforts should not only be put into improving the RET system itself but also into developing signal-detecting devices with increased sensitivity to advance the applications of RET biosensors. Recent years have seen some practical detecting devices being developed. In addition to the commonly used well-scanning plate readers, charge-coupled device (CCD) cameras are used in BRET detection for faster and simpler detection process [101]. It is conducive to high-throughput detection. Microfluidic has also been applied to RET signal detection, especially in the development of portable detection devices to obtain rapid results using fewer samples [65,102,103,104]. The use of smartphones to detect RET signals is also a future direction. Simple methods have been developed to detect RET signals using smartphones [65,102,105,106,107,108]. FRET and BRET will provide more support and inspiration to researchers in future.

## Figures and Tables

**Table 1:** Classic CTZ-consuming luciferase BRET systems.

System	Donor	Acceptor	Substrate	Features	Ref
BRET1	Rluc/Rluc8	EYFP	Coelenterazine-h		[39]
BRET2	Rluc	GFP2/GFP10	DeepBlueC (bisdeoxycoelenterazine, coelenterazine 400a, di-dehydro coelenterazine)	Enlarged separation of donor and acceptor emission spectra, higher signal resolution	[40,41]
BRET3	Rluc8	mOrange	Coelenterazine-h	Longer wavelengths of the emission light and weaker attenuation of biological tissue	[41,45]
eBRET	Rluc	eYFP	EnduRen	Prolonged detection timescale from minutes to hours, enhanced luminescence intensity	[42,43,44]
BRET3.1	Rluc8	mOrange	Coelenterazine-v		[46]
BRET4	Rluc8	TagRFP	Coelenterazine-h		[47]
BRET4.1	Rluc8	TagRFP	Coelenterazine-v		[46]
BRET5	Rluc8.6	TagRFP	Coelenterazine-h		[46]
BRET6	Rluc8.6	TurboFP635	Coelenterazine-h		[46]
BRET6.1	Rluc8.6	TurboFP636	Coelenterazine-v		[46]
BRET7	Rluc8	TurboFP637	Coelenterazine-v		[47]
BRET8	Rluc8.6	TurboFP638	Coelenterazine-h		[47]
BRET9	ALuc23	FP, such as mCherry	Coelenterazine	A conceptually unique ligand-activatable BRET system	[48]

**Table 2:** FRET- and BRET-based biosensors for bioassay and diagnosis.

Principle	Analyte	Donor/Acceptor	Source of Sample	LOD/Linear Range	Ref.
FRET	Exosome	Cy3/MXenes		1.4 × 10^3^ particles mL^−1^	[57]
FRET	EpCAM	QDs/Cy3	Serum	600 pM	[58]
FRET	Dopamine	FAM/TAMRA	Serum		[59]
FRET	Isoleucine	ECFP/Venus	Live cells		[60]
FRET	Polysialic acid (PSA)	SQDs/Cy5	Serum	0.63 pM, 10 pM to 1 μM	[61]
FRET	cGMP	CFP/Venus, T-Sapphire/Dimer2	Live cells		[62]
FRET	Glutathione (GSH) and SO_2_	CM/BP	Live cells	75 μM for GSH and 0.16 μM for SO_2_	[63]
BRET	Small molecule drugs	NanoLuc/Cy3	Whole blood		[66]
BRET	Antibodies such as those against HIV1-p17, hemagglutinin (HA), and dengue virus type I	NanoLuc/mNeonGreen	Plasma	10 pM	[67,68,69]
BRET	Antibodies such as antiHIV1, anti-HA, and anti-DEN1	NanoLuc/mNeonGreen	Whole blood	LODs of 2.8 nm, 7.1 nm, and 19.3 nm for anti-HIV1, anti-HA, and anti-DEN1, respectively	[70]
BRET	Antigen such as osteocalcin/BGP	NanoLuc/maleimide dye in Q-body	Solution	0.11 nM	[72]
BRET	Metabolites	NanoLuc/Cy3	Whole blood		[73]
BRET	miRNA	NanoLuc/mNeonGreen	Serum		[74]

**Table 3:** FRET- and BRET-based biosensors for environmental applications.

Principle	Analyte	Donor/Acceptor	Source of Samples	LOD/Linear Range	Ref.
FRET	Ochratoxin A (OTA)	QDs of different sizes	Agro-products	5 pg/mL	[88]
FRET	Aflatoxin B1 (AFB1)	QDs/AuNPs	Agro-products	3.4 nM, 10–400 nM	[89]
FRET	Organophosphorus pesticides (Ops)	CQDs/AuNPs	Tap and river water samples	0.05 μg L^−1^, 0.05–50 μg L^−1^	[90]
FRET	Hg^2+^	UCNPs/GNPs	Tap water and milk samples	60 nM, 0.2–20 μM	[91]
FRET	Pb^2+^	CDs/Au NRs	Tap water and river water samples	0.05 μM, 0 to 155 μM	[92]
FRET	Bacteria	UCNPs/AuNPs	Food and water samples	3 cfu/mL, 5–10^6^ cfu/mL	[93]
BRET	Ca^2+^/Mg^2+^	NanoLuc/Venus	Water		[94]

**Table 4:** Recently developed FRET- and BRET-based whole-cell sensors for dynamic analysis of metabolic flux.

Target Molecule	Donor/Acceptor	Sensor	Kd	LOD/Linear Range	Host	Ref.
Lysine	CFP and YFP	LAO	97μM		*Escherichia coli* and *Saccharomyces cerevisiae*	[95]
Leucine	CFP and YFP	LivK	192 mM, 510 mM, 50 mM, and 105 mM, respectively, in different types	900 mM, 10–1000 mM, 8.0–500 mM, and 150–800 Mm, respectively, in different types	*Escherichia coli* and *S. cerevisiae*	[96]
(+)-Catechin	ECFP and Venus	fraa-3	139 µM		*Escherichia coli*	[97]
α-Tocopherol	ECFP and Venus	TTPA	100 µM		*Escherichia coli*	[98]
Ajmalicine	ECFP and Venus	CYP2D6			*Catharanthus roseus* (L.) G. Don	[99]
N-acetyl-5-neuraminic acid (NeuAc)	ECFP and Venus	SiaP	∼157 µM		*Escherichia coli*	[100]
